# Use of Prophylactic Methylxanthines to Prevent Extubation Failure in Preterm Neonates with a Birth Weight of 1250–2499 g: A Propensity Score-Matched Analysis

**DOI:** 10.3390/jcm14113856

**Published:** 2025-05-30

**Authors:** Pachima Suwankomonkul, Anucha Thatrimontrichai, Pattima Pakhathirathien, Manapat Praditaukrit, Gunlawadee Maneenil, Supaporn Dissaneevate, Chamaiporn Trangkhanon, Neeracha Phon-in

**Affiliations:** Division of Neonatology, Department of Pediatrics, Faculty of Medicine, Prince of Songkla University, Songkhla 90110, Thailand; spachima@medicine.psu.ac.th (P.S.); ppattima@medicine.psu.ac.th (P.P.); manapat.p@psu.ac.th (M.P.); mgunlawa@medicine.psu.ac.th (G.M.); dsupapor@medicine.psu.ac.th (S.D.); tchamaip@medicine.psu.ac.th (C.T.); pneerach@medicine.psu.ac.th (N.P.-i.)

**Keywords:** airway extubation, aminophylline, bronchopulmonary dysplasia, caffeine, methylxanthine, neonatal intensive care unit, non-invasive ventilation, premature infant, nasal high-frequency oscillatory ventilation, nasal intermittent positive pressure

## Abstract

**Background/Objectives:** Preterm neonates with a birth weight (BW) of 500–1250 g who receive prophylactic methylxanthine have a lower rate of bronchopulmonary dysplasia and neurodevelopmental disability than their counterparts. In a meta-analysis of previous studies (published during 1985–1993, with no routine continuous positive airway pressure), extubation failure rates in preterm neonates with BW < 2500 g who received and did not receive methylxanthine were 25.0% and 50.6%, respectively (risk difference, −0.27; 95% confidence interval [CI], −0.39 to −0.15). However, no study to date has assessed the effects of prophylactic methylxanthine use on endotracheal extubation in infants weighing 1250–2499 g until now. **Methods**: First-time extubation was compared between 1:1 propensity score-matched methylxanthine and non-methylxanthine groups from a retrospective cohort of 541 neonates (born during 2014–2024). **Results**: The domains from the overall cohort and propensity-matched data included 541 and 192 neonates, respectively. In the propensity score-matched sample, the mean gestational age and BW were 30.9 ± 1.9 weeks and 1584 ± 273 g, respectively. The median 5-min Apgar score was 9 (range of 7–9). Extubation failure within 7 days occurred in 10 (10.4%) and 13 (13.5%) neonates in the methylxanthine (*n* = 96) and non-methylxanthine (*n* = 96) groups, respectively, with a risk difference (95% CI) of −0.03 (−0.12 to 0.06), *p* = 0.50, and hazard ratio (95% CI) of 0.76 (0.33 to 1.72), *p* = 0.51. **Conclusions**: In the current era with new non-invasive ventilation approaches, extubation failure in preterm neonates with a BW of 1250–2499 g is not significantly affected by the use of methylxanthine.

## 1. Introduction

Endotracheal intubation is required in preterm neonates with respiratory failure. Tracheal extubation may be difficult because preterm neonates have poor central and peripheral respiratory drives and diaphragmatic strength. Methylxanthines have routinely been prescribed to treat apnea of prematurity (AOP) in preterm infants since the 1970s and to prevent neurodevelopmental disability, as in the Caffeine for Apnea of Prematurity (CAP) trial in 2006. The CAP trial assigned 2006 infants with a birth weight (BW) of 500–1250 g during the first 10 days of life to receive either prophylactic caffeine or placebo until the infant had tolerated at least 5 consecutive days without the use of positive airway pressure. The median postmenstrual ages at the first and last doses in the CAP trial were 28 and 35 weeks, respectively. Compared with the placebo group, the caffeine group had a lower rate of bronchopulmonary dysplasia (BPD) before discharge [1], lower rate of mortality or survival with neurodevelopmental disability at 18–21 months [2], and higher scores for fine motor coordination and visual abilities at 11 years [3].

Some medications may improve pulmonary function and increase extubation success. Methylxanthines, which include caffeine, theophylline, and aminophylline, stimulate the central respiratory drive and peripheral chemoreceptor activity, increase diaphragmatic contractility, and reduce apnea, thereby improving effective extubation. A meta-analysis reported that methylxanthine treatment in preterm and low-BW (LBW; BW < 2500 g) infants may reduce extubation failure within 7 days (50.6% [45/89] versus 25.0% [27/108]; risk ratio [RR], 0.48; 95% confidence interval [CI], 0.32 to 0.71; risk difference [RD], −0.27; 95% CI, −0.39 to −0.15; number needed to treat, 4; 95% CI, 3 to 7; six trials, 197 infants) [4]. However, this meta-analysis had some limitations [4]. It included publications from the 1980s to 1990s with different ventilators and settings and varied use of non-invasive ventilation (NIV) support (only one study used routine nasal continuous positive airway pressure [nCPAP], while five studies did not routinely use NIV), an overall small sample size of 197 infants, and a varied population (BW < 1.25 [5,6], 1.75 [7], 2 [8], and 2.5 kg [9], or gestational age [GA] < 34 weeks [10]). The methylxanthines used also varied in the meta-analysis and included aminophylline [5,9], caffeine [7,8], and theophylline [6,8,10]. The outcome of each included study was extubation failure at any time [5] or within 5 [6,7] or 7 [9] days.

According to a recent systematic review, extubation was more likely to fail in neonates with a lower GA, BW, and body weight and age at extubation than in their counterparts. Higher levels of respiratory support on the date of extubation, indicated by lower pre-extubation pH and higher pre-extubation mean airway pressure, fraction of inspired oxygen, and pCO_2_, were factors associated with the risk of extubation failure [11]. The incidences of extubation failure in extremely preterm (GA < 28 weeks), very preterm (GA < 32 weeks), and preterm (GA < 37 weeks) infants were 50–60% [12,13], 20–30% [12,13,14], and 26% [14], respectively.

Preterm neonates with a BW < 1250 g and with/without endotracheal intubation have been routinely administered methylxanthine for the prophylactic treatment of BPD, neuroprotection, and the treatment of AOP [1,2,3]. However, no study has assessed the effects of prophylactic methylxanthines on endotracheal extubation in infants weighing 1250–2499 g in the current era with new NIV. Therefore, in this study, we performed a retrospective propensity score-matched analysis of the effects of prophylactic methylxanthine treatment on endotracheal extubation in infants with a BW of 1250–2499 g in Thailand.

## 2. Methods

### 2.1. Study Design and Patient Domains

This retrospective cohort study was performed using the medical records of all neonates admitted to the level IV neonatal intensive care unit of our institution. Data identified from our database from 1 January 2014 to 31 December 2024 included baseline characteristics, comorbidities, and outcomes. This study was approved by the Ethics Committee of the Faculty of Medicine, Prince of Songkla University (REC. 66-525-1-1). Informed consent for participation was not required owing to the retrospective study design.

The inclusion criteria were preterm infants with a BW of 1250–2499 g who underwent first extubation after being intubated. We excluded neonates with major congenital anomalies (including neurological, cardiovascular, pulmonary, gastrointestinal, renal, and chromosomal diseases), those administered therapeutic methylxanthine for apnea before extubation, those who underwent unplanned extubation, those who underwent reintubation for surgery after extubation, those who started methylxanthine within 7 days after extubation and did not receive methylxanthine before extubation, those who were transferred to another hospital with an endotracheal tube, and those who died before extubation.

### 2.2. Ventilatory Care

High-frequency oscillatory ventilation (HFOV) was used as the initial treatment or primary mode for intubated preterm neonates, followed by nasal HFOV (nHFOV), (synchronized) intermittent positive pressure (n[S]IPPV), nCPAP, bilevel positive airway pressure ventilation, or high-flow oxygen cannula as NIV after extubation, depending on the clinical condition of the infant and the decision of the attending staff. From 2014 to 2024, intravenous (IV) aminophylline was routinely administered to preterm infants with a BW < 1250 g or infants with apnea who were switched to oral caffeine when they could tolerate enteral feeding.

### 2.3. Exposure, Comorbidities, and Outcomes

The methylxanthine group (MX group) included all neonates who received IV aminophylline and oral caffeine if oral feeding could be tolerated before extubation. The loading dose of aminophylline was 8 mg/kg via IV infusion (if their extubation was planned for <3–5 days after aminophylline administration), and the maintenance dose was 1.5–3 mg/kg IV administered every 8–12 h. The maintenance dose of the caffeine base was 2.5–5 mg/kg administered orally every 24 h. The non-methylxanthine group (NX group) included neonates who did not receive aminophylline or caffeine before extubation.

Ventilator-associated pneumonia was diagnosed according to the National Healthcare Safety Network guidelines for infants aged <1 year [15] by a pediatrician and pediatric radiologist. Patent ductus arteriosus (PDA) and persistent pulmonary hypertension of the newborn were diagnosed in newborns on the basis of clinical and echocardiographic criteria before extubation. Mean airway pressure and fraction of inspired oxygen were recorded before extubation. Arterial pH and pCO_2_ were measured within 12–24 h of extubation, and data from the time point nearest to extubation were used for analysis. Sedative drug use was defined as the use of IV midazolam within 24 h before extubation. The new modes of NIV were defined as nHFOV and n(S)IPPV support after extubation. Nasal bilevel positive airway pressure ventilation, nCPAP, and high-flow nasal cannula were not considered new modes of NIV.

Extubation failure was defined as the necessity of reintubation within 7 days of extubation. Moderate or severe BPD was defined as the need for supplemental oxygen for at least 28 days until the infant was 36 weeks’ postmenstrual age or at 56 days of life for babies born before or after 32 weeks’ GA, respectively, or until discharge, whichever occurred first [16]. Severe neurological injury was defined as intraventricular hemorrhage grades 3–4 and/or periventricular leukomalacia. Infants who had been treated with either laser surgery or intraocular anti-vascular endothelial growth factor injections were classified as having treated retinopathy of prematurity. Cranial ultrasonography and eye examinations were only performed in neonates with relevant indications.

Tachycardia was defined as a heart rate > 180 beats/min from 24 h after starting methylxanthine until 5 days after the discontinuation of the medication in the MX group or at any time during admission in the NX group. Seizures were documented when they occurred after methylxanthine administration and had no secondary cause. Feeding intolerance was defined as the inability to digest breast or formula milk, for example, vomiting, abdominal distension, large volumes of gastric residuals several times, and then stopping oral feeding for at least 48 h. Necrotizing enterocolitis was diagnosed on the basis of Bell’s criteria and clinical and radiographic evidence, and stages II–III were considered relevant [17].

### 2.4. Sample Size Calculation

Previous meta-analyses [4,18] indicated that methylxanthines can decrease the rate of extubation failure in LBW infants from 50.6% (45/89) to 25.0% (27/108) [5,6]. We calculated that, for the comparison of two independent proportions, using a significance level of <5% and 80% power, a sample of 131 neonates per group was required to detect differences in extubation failure. We reviewed the overall cohort data to obtain at least double this number per group (total: 524 neonates).

### 2.5. Statistical Analysis

The STATA software (version 17; StataCorp LLC, College Station, TX, USA) was used to compare the baseline characteristics, comorbidities, and outcomes between the MX and NX groups. Categorical variables are presented as percentages and were compared using the chi-squared or Fisher’s exact test. The Shapiro–Wilk test was used to determine the normality of data distribution for continuous variables. Parametric variables are presented as mean ± standard deviation, and a *t*-test was used to compare unpaired samples. Non-parametric continuous variables are presented as median (interquartile range), and the Mann–Whitney *U* test was used to compare unpaired samples.

Independent variables were chosen on the basis of biologic plausibility, with either *p* < 0.2 in the univariable analysis or significant variables from previous studies [11]. The adjusted risk difference (RD) and hazard ratio (HR) with 95% CIs were computed for variables independently associated with the MX and NX groups in a multivariable regression model.

### 2.6. Propensity Score Matching

The mapsm command in STATA matches the propensity scores, predicted from binary logistic regression, between groups, and indicates the likelihood of each participant being assigned to one of the other treatment arms. The propensity scores (probability of 0–1.0) derived from binary logistic regression are divided into 10 strata: 0–0.1, 0.1–0.2, etc., until 0.9–1.0. Study participants from either of the two arms were sampled to match the opposite arm within the same propensity score strata at a 1:1 ratio. The command also reran the matching process by an iteration round of 200 and reported the best post-matched contrast groups that were best balanced, as determined by the standardized difference. The best seed-setting number (seed 1243 in this study) was reported to obtain the best post-matching cohort.

**Figure 1:**
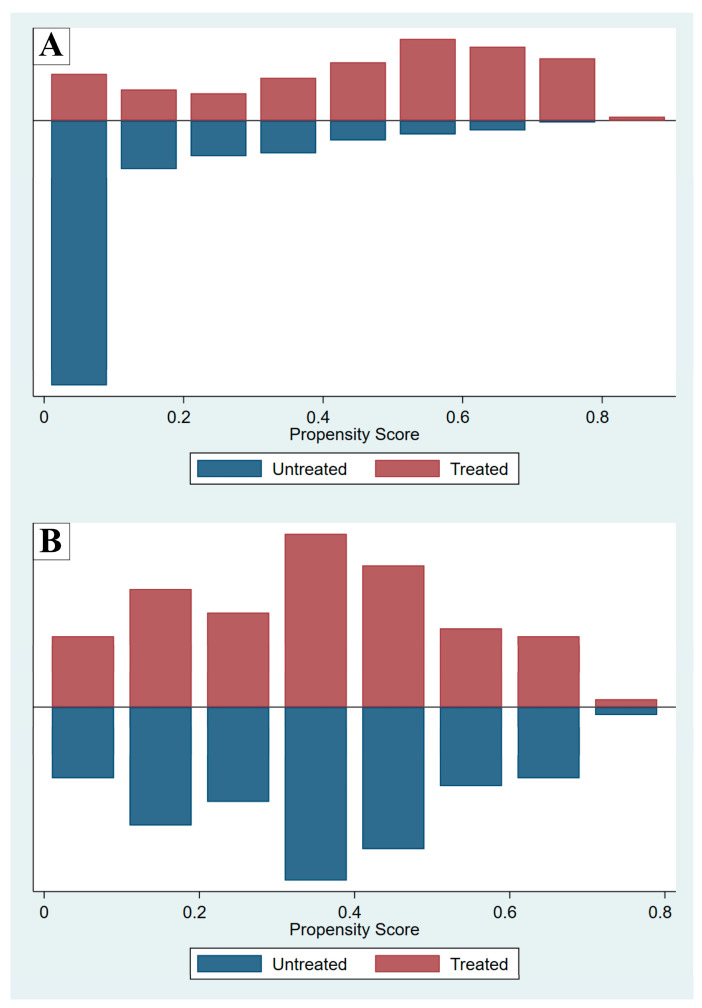
Propensity scores before (**A**) and after (**B**) matching, comparing the methylxanthine-treated (Treated) and non-methylxanthine-treated (Untreated) groups.

Propensity score matching was performed to reduce confounders by indication and selection biases inherent to observational studies. The propensity score, which in this study was the predicted probability of receiving methylxanthine, was calculated using baseline covariates (a clinician’s decision to prescribe methylxanthine to an intubated preterm infant based on clinical indications: GA, BW, and 5-min Apgar score) in a logistic regression model. After propensity score matching, a matched cohort of patients with covariates balanced between the groups was assessed using standardized differences before and after matching, with a standardized difference < 0.1–0.2 indicating insignificant differences between the groups. A comparison of the propensity scores in the MX and NX groups before and after matching is shown in Figure 1.

## 3. Results

**Figure 2:**
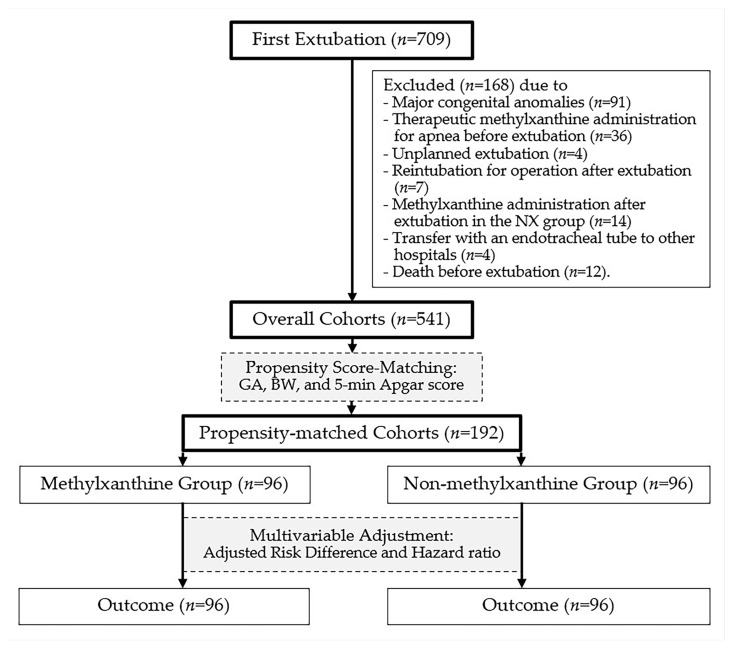
Study patient data flow diagram.

From 2014 to 2024, the number of extubation events in preterm infants with a BW of 1250–2499 g at our institution was 730. First extubation events accounted for 709 neonates, of whom 168 were excluded owing to major congenital anomalies (*n* = 91), therapeutic methylxanthine administration for apnea before extubation (*n* = 36), unplanned extubation (*n* = 4), reintubation for operation after extubation (*n* = 7), methylxanthine administration after extubation in the NX group (*n* = 14), transfer with an endotracheal tube to other hospitals (*n* = 4), and death before extubation (*n* = 12) (Figure 2). Finally, data were analyzed for the overall (*n* = 541) and propensity-matched (*n* = 192) cohorts (Table 1).

In the overall cohort (*n* = 541), the mean GA and BW were 32.5 ± 2.2 weeks and 1842 ± 364 g, respectively. The median (interquartile range) 5-min Apgar score was 9 (8–9). Of the neonates, 306 (56.6%) were male, 409 (75.6%) were delivered via cesarean section, 126 (23.3%) involved multifetal pregnancies, and 203 (37.5%) were treated using new modes of NIV.

In the propensity score-matched data (*n* = 192), the mean GA and BW were 30.9 ± 1.9 weeks and 1584 ± 273 g, respectively. The median (interquartile range) 5-min Apgar score was 9 (7–9). Of the neonates, 107 (55.7%) were male, 144 (75.0%) were delivered via cesarean section, 44 (22.9%) involved multifetal pregnancies, and 93 (48.4%) were treated using new modes of NIV.

Baseline characteristics and risk factors for extubation failure of the MX and NX groups are shown in Table 2. RD and hazard regression analyses showed that extubation failure did not differ significantly between the groups after adjusting for factors with *p* < 0.2 in univariable analyses (inborn neonates, multifetal gestation, cesarean section, respiratory distress syndrome, surfactant administration, persistent pulmonary hypertension, PDA, body weight, postnatal age at extubation, mean airway pressure, fraction of inspired oxygen, and use of new modes of NIV).

**Figure 3:**
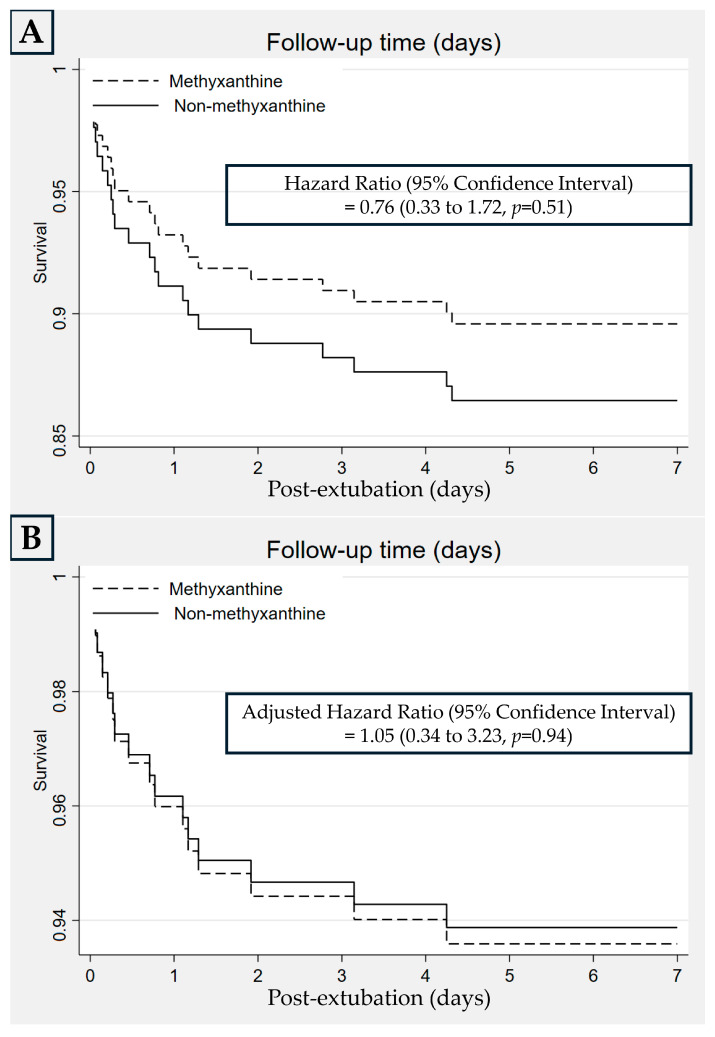
Kaplan–Meier curves for the rate of extubation failure between the methylxanthine and non-methylxanthine groups by univariable (**A**) and multivariable (**B**) analyses.

Extubation failure within 7 days in the MX and NX groups occurred in 10 (10.4%) and 13 (13.5%) neonates, respectively, with an RD (95% CI) of −0.03 (−0.12 to 0.06), *p* = 0.50, and an adjusted RD (95% CI) of 0.01 (−0.10 to 0.13), *p* = 0.83 (Table 3). For this comparison, the HR (95% CI) was 0.76 (0.33 to 1.72), *p* = 0.51, and the adjusted HR (95% CI) was 1.05 (0.34 to 3.23), *p* = 0.94 (Figure 3). In the subgroup analysis, extubation failure within 7 days was not significantly different between the MX and NX groups in neonates with BWs of 1250–1999 and 1250–1499 g (Table 3). In the MX group, extubation failed in 10 infants because of frequent clinical apneic episodes (*n* = 2; time to reintubation: 1 and 6 h), atelectasis (*n* = 2; time to reintubation: 18 and 75 h), poor respiratory effort (*n* = 1; time to reintubation: 11 h), PDA (*n* = 3), vocal cord edema (*n* = 1), and secretion obstruction (*n* = 1). In the NX group, extubation failed in 13 infants because of frequent clinical apneic episodes (*n* = 3; time to reintubation: 1, 1, and 66 h), atelectasis (*n* = 2; time to reintubation: 19 and 103 h), poor respiratory effort (*n* = 2; time to reintubation: 2 and 46 h), vocal cord edema (*n* = 3), PDA (*n* = 2), and sepsis (*n* = 1). If the causes of extubation failure were consolidated into a composite consisting of frequent apnea, atelectasis, and poor respiratory effort (effects of methylxanthine), five and seven neonates had extubation failure in the MX and NX groups, respectively.

The incidence of moderate or severe BPD and severe neurological injury was lower in the MX than in the NX group; however, the difference was not statistically significant (RD = 0.01 and 0.01, respectively; Table 3). No deaths, seizures, or treated retinopathy of prematurity occurred in either group. The median duration of methylxanthine therapy after extubation was 9 (4–22) days. The rate of feeding intolerance in the MX group was higher than that in the NX group (RD = 0.13); however, this difference was not statistically significant after adjustment for covariates in the multivariable model.

## 4. Discussion

In this study, we investigated whether extubation failure within 7 days in preterm neonates with a BW of 1250–2499 g was affected by methylxanthine administration. Our findings show that extubation failure within 7 days occurred in 10 (10.4%) and 13 (13.5%) neonates with and without methylxanthine treatment. These results indicate that, when using new NIV approaches, extubation failure in preterm neonates with a BW of 1250–2499 g is not significantly affected by the use of methylxanthine.

Methylxanthines, particularly caffeine, should routinely be administered to preterm infants with a BW < 1250 g, irrespective of whether they are endotracheally intubated [1,3]. Previous meta-analyses [4,18] have indicated that methylxanthines can decrease the rate of extubation failure in LBW infants from 50.6% (45/89, NX) to 25.0% (27/108, MX) and from 40.0% (20/50, NX) to 23.9% (17/71, MX) if two randomized controlled trials (RCTs) in neonates with a BW < 1250 g are excluded (Table 4) [5,6]. The current propensity score-matched study showed no significant difference in extubation failure in preterm infants with a BW 1250–2499 g between the MX (10.4%, 10/96) and NX (13.5%, 13/96) groups. If a high rate of extubation failure (40–50%) is experienced in LBW infants, the administration of any methylxanthine before extubation should be considered (considering the loading dose, if this is administered < 3–5 days before extubation). Compared with nCPAP, the new modes of NIV, including nHFOV and n(S)IPPV, reduce reintubation within 7 days after extubation [19,20,21,22]. In a neonatal intensive care unit setting with high rates of utilization of new NIV modes (37.5–48.4%, similar to that in our unit) for routine post-extubation care, methylxanthine use may not make a difference in extubation failure in a setting where this rate is already low (13.5%).

In previous meta-analyses, six RCTs compared methylxanthine use in preterm neonates [4,18]. The outcomes in four of these RCTs were considered relevant, as two RCTs studied neonates with a BW < 1250 g [5,6]. These four RCTs were published in 1985 [10], 1992 [7], and 1993 [9], while one study was a conference proceeding reported in 1991 [8]. nCPAP was applied as routine post-extubation care [8], applied only in some cases [7], and not mentioned in two RCTs [9,10]. In the present study, the percentage of cases in whom the new NIV modes (nHFOV and n[S]IPPV) were used after extubation was 37.5–48.4%, whereas the remaining cases received nCPAP. The mean BWs in the intervention group from the previous and current studies were 1389 g [7], 1415 g [9], 1448 g [10], and 1583 g (this study). The postnatal ages at extubation in the intervention group were 2.0 days (this study), 3.4 days [9], 3.8 days [10], and 5 days [7]. The durations of methylxanthine administration were at least 48 h [10], 5 days [9], 7 days [7], and 9 days (this study) after extubation. Times to extubation failure were 48 h [10], 5 days [6,7], and 7 days (this study) [9]. However, the baseline characteristics (BW and postnatal age) and duration of methylxanthine use in this study were similar to those used in previous studies. The different eras of the study (in which primary HFOV or the new modes of NIV were used) may have affected the primary outcome. The extubation failure rate was high (40.0% [NX] to 23.9% [MX]) in the four RCTs included in the meta-analyses and low (13.5% [NX] to 10.4% [MX]) in the present study (Table 4).

This study has several strengths: First, a propensity score-matched analysis of database data provides the best available evidence, given the challenges in conducting or replicating an RCT in preterm infants (fragile participants). We had a larger sample size (541 cases, 192 cases after propensity score matching) than that used in previous studies (*n* = 18 [7], 20 [9], 38 [10], and 45 [8] cases). Second, we attempted to minimize the effect of confounders by using a propensity score-matched analysis. Some variables, including GA, BW, and the 5-min Apgar score, may determine or indicate the need for methylxanthine use after birth. Multivariable regression analyses (RD and hazard regression) were used to adjust for potential confounders or co-interventions. Third, this study fills the knowledge gap for clinical implications in intubated preterm neonates with a BW of 1250–2499 g in the era of new NIV modes. However, further RCTs with larger sample sizes are warranted.

Nevertheless, caution should be exercised when interpreting and generalizing the results of this study, as it has some limitations. First, we collected long-term data from a database to maximize the sample size, but co-interventions may have been involved in the long term. Invasive HFOV was used as the routine primary mode of ventilation after intubation and methylxanthine administration (IV aminophylline and oral caffeine) over the 11-year study period. nHFOV [23] and nSIPPV [20,24] have been implemented for post-extubation support at our center since 2013 and 2020, respectively. In recent meta-analyses, compared with nCPAP, both new modes of NIV decreased the rate of reintubation in preterm infants [19,21,22]. However, no other co-intervention, particularly in terms of respiratory care, has changed over time. Second, despite our attempts to control for confounders by indication (using propensity score matching), residual confounders may have remained, given the limited data on staff preferences (methylxanthine use, interface, and settings of NIV due to lack of local guidelines). Third, although the sample size in the propensity score matching (192 neonates) was larger than that in the previous meta-analysis (121 neonates), the post hoc power and sample size calculations were 9.7% and 1716 neonates per arm, respectively. Fourth, methylxanthine (IV aminophylline and an oral caffeine base) has been administered for more than 20 years, because IV caffeine has been available in our country since 2019. However, IV caffeine is still unavailable in our unit and is not covered by government healthcare in our country. Finally, the side effects were similar in both groups; however, tachycardia may have been under-reported by definition (persistent for more than 24 h), and methylxanthine was withheld or decreased in some neonates with tachycardia. Moreover, the therapeutic drug monitoring was not routinely performed in the MX group.

## 5. Conclusions

In previous meta-analyses, methylxanthine use before extubation decreased the rate of extubation failure in preterm and LBW neonates born during 1985–1993, unlike nCPAP during routine post-extubation care. Over the past decade, new NIV modes have been used in clinical practice. In the NX group, the reintubation rate decreased from 40% to 13.5%. Prophylactic methylxanthine use in preterm neonates with a BW 1250–2499 g may not provide substantial additional benefit if the extubation failure rate has reached 13.5% by implanting the new ventilatory strategies, i.e., avoiding intubation and applying gentle invasive ventilation (using primary HFOV mode), using aggressive extubation, and providing full respiratory support with NIV (using nHFOV and n[S]IPPV modes). The clinical implications may vary with the incidence of extubation failure in each local hospital. Further RCTs with larger sample sizes, investigating both routes of caffeine use, and new modes of NIV, are required.

## Figures and Tables

**Table 1:** Baseline characteristics of the methylxanthine (MX) and non-methylxanthine (NX) groups before and after propensity score matching.

Baseline Characteristic	Overall Cohort (*n* = 541)				Propensity Score-Matched Cohort (*n* = 192)			
	MX (*n* = 110)	NX (*n* = 431)	Standardized Difference	*p*-Value	MX (*n* = 96)	NX (*n* = 96)	Standardized Difference	*p*-Value
Gestational age, weeks	30.7 ± 1.8	32.9 ± 2.1	1.16	<0.01	30.9 ± 1.9	31.0 ± 2.0	0.02	0.88
Birth weight, g	1551 ± 280	1916 ± 346	1.16	<0.01	1583 ± 285	1586 ± 263	0.01	0.95
5-min Apgar score	9 (7–9)	9 (8–9)	0.15	0.16	9 (7–9)	9 (7–9)	0.004	0.94

**Table 2:** Baseline characteristics and risk factors for extubation failure in the methylxanthine (MX) and non-methylxanthine (NX) groups.

Baseline Characteristic	MX (*n* = 96)	NX (*n* = 96)	*p*-Value
Inborn neonate, *n* (%)	95 (99.0)	72 (75.0)	<0.01
Multifetal gestation, *n* (%)	28 (29.2)	16 (16.7)	0.04
Cesarean section, *n* (%)	79 (82.3)	65 (67.7)	0.02
Male sex, *n* (%)	56 (58.3)	51 (53.1)	0.47
Respiratory distress syndrome, *n* (%)	88 (91.7)	56 (58.3)	<0.01
Surfactant administration, *n* (%)	50 (52.1)	31 (32.3)	0.01
Ventilator-associated pneumonia, *n* (%)	2 (2.1)	3 (3.1)	0.65
Patent ductus arteriosus, *n* (%)	43 (44.8)	24 (25.0)	<0.01
Persistent pulmonary hypertension, *n* (%)	1 (1.0)	5 (5.2)	0.10
**Risk Factors on Date of Extubation**	**MX (*n* = 96)**	**NX (*n* = 96)**	***p*-Value**
Body weight, g ^a^	1559 ± 296	1722 ± 378	<0.01
Postnatal age, days ^b^	2 (1–5)	3 (1–15)	0.02
Mean airway pressure, cmH_2_O ^a^	7.5 ± 1.2	7.1 ± 1.4	0.08
Fraction of inspired oxygen ^a^	0.27 ± 0.06	0.29 ± 0.07	0.16
Arterial pH ^a^	7.36 ± 0.07	7.35 ± 0.06	0.26
Arterial pCO_2_, mmHg ^b^	38.5 (32.8–44.0)	39.8 (34.9–45.5)	0.22
Sedative drug use, *n* (%)	5 (5.2)	2 (2.1)	0.25
New modes of non-invasive ventilation, *n* (%)	60 (62.5)	33 (34.4)	<0.01

^a^ Mean ± standard deviation or ^b^ median (interquartile range).

**Table 3:** Results of the univariable and multivariable analyses of outcomes in the methylxanthine (MX) and non-methylxanthine (NX) groups.

Outcome	MX (*n* = 96), *n* (%)	NX (*n* = 96), *n* (%)	Univariable Analysis		Multivariable Analysis	
			RD (95% CI)	*p*-Value	Adjusted RD (95% CI) ^a^	*p*-Value
Extubation failure within 7 days						
Birth weight 1250–2499 g	10/96 (10.4)	13/96 (13.5)	−0.03 (−0.12, 0.06)	0.50	0.01 (−0.10, 0.13)	0.83
Birth weight 1250–1999 g	10/88 (11.4)	12/88 (13.6)	−0.02 (−0.12, 0.08)	0.65	0.04 (−0.08, 0.16)	0.50
Birth weight 1250–1499 g	6/48 (12.5)	6/48 (12.5)	0 (−0.13, 0.13)	1.00	0.04 (−0.16, 0.23)	0.71
Moderate or severe bronchopulmonary dysplasia	7 (7.3)	8 (8.3)	−0.01 (−0.09, 0.07)	0.79	−0.04 (−0.14, 0.06)	0.40
Severe neurological injury ^b^	7/73 (9.6)	6/57 (10.5)	−0.01 (−0.11, 0.10)	0.86	0.05 (−0.08, 0.17)	0.44
Feeding intolerance	17 (17.7)	5 (5.2)	0.13 (0.04, 0.21)	0.01	0.10 (−0.03, 0.22)	0.12
Necrotizing enterocolitis	6 (6.3)	6 (6.3)	0 (−0.07, 0.07)	1.00	0.02 (−0.07, 0.11)	0.66
Spontaneous intestinal perforation ^c^	0 (0)	2 (2.1)	–		–	
Tachycardia ^c^	1 (1.0)	3 (3.1)	–		–	

Values are presented as frequency (percentage) or RD (95% CI), risk difference (95% confidence interval). ^a^ Adjusted for inborn neonates, multifetal gestation, cesarean section, respiratory distress syndrome, surfactant administration, persistent pulmonary hypertension, patent ductus arteriosus, body weight and postnatal age on the date of extubation, mean airway pressure, fraction of inspired oxygen, and new modes of non-invasive ventilation. ^b^ Only neonates with indications. ^c^ The rates of incidence were <5%.

**Table 4:** Comparison of extubation failure in preterm infants between the methylxanthine and non-methylxanthine groups from previous studies and the present study.

Study Year	Country	Population	Methylxanthine	Extubation Failure (Intervention vs. Control)	Note
1985 [10]	UK	<34 weeks	Theophylline	2/18 vs. 8/20	BW 1448 g in intervention
1985 [6]	US	<1250 g	Theophylline	5/14 vs. 10/11	CPAP after extubation
1987 [5]	US	<1250 g	Aminophylline	5/23 vs. 15/28	No CPAP after extubation
1991 [8]	-	<2000 g	Caffeine or Theophylline	8/31 vs. 4/14	Proceeding
1992 [7]	Spain	<1750 g	Caffeine	4/12 vs. 6/6	BW 1389 g in intervention; some CPAP after extubation
1993 [9]	Canada	<2500 g	Aminophylline	3/10 vs. 2/10	BW 1415 g in intervention
Meta-analyses of all trials [5,6,7,8,9,10]				27/108 vs. 45/89	25.0% vs. 50.6%
Some trials [7,8,9,10] excluded infants with a BW < 1250 g [5,6]				17/71 vs. 20/50	23.9% vs. 40.0%
Present study		1250–2499 g	Aminophylline, Caffeine	10/96 vs. 13/96	Propensity score matching of 541 neonates

BW, birth weight; CPAP, continuous positive airway pressure; vs., versus.

## Data Availability

The raw data supporting the conclusions of this study will be made available by the authors upon request. The data are not publicly available due to privacy concerns.

## Abbreviations

The following abbreviations are used in this manuscript:

AOP	apnea of prematurity
CAP	Caffeine for Apnea of Prematurity
BW	birth weight
BPD	bronchopulmonary dysplasia
LBW	low-birth weight
NIV	non-invasive ventilation
nCAP	nasal continuous positive airway pressure
GA	gestational age
HFOV	high-frequency oscillatory ventilation
nHFVO	nasal high-frequency oscillatory ventilation
n(S)IPPV	nasal (synchronized) intermittent positive pressure
IV	intravenous
MX group	methylxanthine group
NX group	non-methylxanthine group
PDA	patent ductus arteriosus
RD	risk difference
HR	hazard ratio
CI	confidence interval
